# Oncoplastic Breast Surgery: What, When and for Whom?

**DOI:** 10.1007/s12609-016-0212-9

**Published:** 2016-05-03

**Authors:** R. Douglas Macmillan, Stephen J. McCulley

**Affiliations:** Nottingham Breast Institute, Nottingham, NG5 1PB UK

**Keywords:** Oncoplastic breast surgery, Breast-conserving surgery, LICAP, Therapeutic mammaplasty

## Abstract

Oncoplastic surgery is integral to all breast cancer surgeries. The use of an aesthetic approach to breast conservation or mastectomy greatly enhances the range of options that can be offered to women with breast cancer and facilitates better outomes from it. It should be the standard of care. However, a structured approach to selecting appropriate techniques is essential, and although many operative procedures are reported, this article sets out to describe a set of principles and an algorithm by which the what, when and for whom for oncoplastic surgery can be defined.

## Introduction

Oncoplastic breast surgery was a term originally coined in the 1980s to reflect the integration of chemotherapy and radiotherapy planning with conservative forms of breast surgery for more advanced disease. Its aim was to achieve better aesthetic and quality of life outcomes compared to traditional approaches with less morbidity. The origins were therefore multidisciplinary, and early developments in oncoplastic surgery served to show that even in locally advanced disease, obvious deformity was avoidable [1]. The term is now used ubiquitously to represent any surgery that aims to maintain quality of life and an acceptable breast appearance whilst at the same time being uncompromising on oncological effectiveness. Obviously, the latter always remains the primary goal, but attention to the consequences of surgical treatment, both short and long term, not just the effectiveness of it, rightly parallels advances in breast cancer survival. In other words, oncoplastic surgery is surgery that is considerate to what we leave women to live with for the rest of their lives and should be an integral part of treatment for all women with breast cancer. Breast cancer has so many negative connotations and insults to a woman’s sense of being whole that it seems startlingly obvious that if the negative impact of surgery can be reasonably mitigated then it should.

Obviously, the first major step towards offering greater choice in the aesthetic outcome of breast cancer surgery was the widespread use of breast-conserving surgery and avoidance of mastectomy where appropriate. Offering even greater choice of operative procedure in this regard and expanding the indications for breast conservation as well as improving the outcomes of it are key aims of oncoplastic surgery. Many techniques of oncoplastic breast-conserving surgery have been described, but in general their evolution has followed three main paths: the use of breast reduction techniques, initially to remove cancers that were located in areas of the breast that could be removed as part of a standard reduction technique but later to include the use of modified techniques to allow resection of any part of the breast; the use of volume replacement techniques, initially using variations on LD flaps but later local perforator-based flaps; and the use of various techniques to allow en bloc closure of breast defects, from simple patterns of skin reduction to modifications of cosmetic mastopexies [2–8]. However, oncoplastic breast surgery is not just about breast conservation. Applying oncoplastic principles to mastectomy and reconstruction is just as important. The ability to perform an oncoplastic mastectomy is a basic requirement of any oncoplastic surgeon, whether they be breast oncology or plastics based. Understanding and applying the range of mastectomy techniques available and how they best apply to different oncological and reconstruction options will give the best potential outcomes.

Essentially, all breast cancer surgery needs to be viewed as oncoplastic. Here, the politics of breast cancer surgery service delivery has provided strong headwinds hindering many. This article does not intend to address these issues but suffice them to say that essentially, they need to be questioned why anyone with a general surgery background without aesthetic skills would operate on the breast and why anyone with a plastic surgery background without oncologic skills or knowledge would operate on a breast cancer patient. Having said that, breast surgery will always require a mixture of oncological, plastic, and microsurgery skills provided by a team of specialists, and the ability to provide this team with the ideal skill base may be aspirational rather than possible in many parts of the world. The point is more that ideally any surgeon performing breast cancer-related surgery requires a minimum skill set, and this has to include knowledge of all and ability in many of the techniques that used to reside solely in the parent specialties of general surgical oncology or plastic surgery.

Therefore, even the traditional simple mastectomy needs to be performed with thought and planning. The use of contralateral breast reduction with unilateral simple mastectomy in extremely ptotic or large-breasted patients has nothing to do with reconstruction but everything to do with an oncoplastic approach and quality of life. Creating low-lying scars, flat surfaces for prosthesis and avoiding dog-ears will improve outcomes even without reconstruction. Different skin and nipple-sparing mastectomy techniques can be selected depending upon patient morphology, breast ptosis and type of reconstruction planned [9]. Performing an oncoplastic mastectomy is a much more difficult skill to acquire than many assume; it is the basis of any good reconstruction and inadequately performed, and it is also the basis of most early complications [10]. Correctly applied, the ideal mastectomy and immediate reconstruction has enabled a dramatic improvement in quality of outcome and crucial to this is the concept that the reconstruction itself needs to be seen as an integral part of mastectomy planning. There are now very few contraindiactions for immediate breast reconstruction in women who wish one, with delayed reconstruction often being recommended for logistical reasons or because of dogmatic and out-dated approaches to managing the challenges posed by post-mastectomy radiotherapy. Reconstruction options need to be considered early, whether they be immediate or delayed, and surgery planned appropriately and considerately from the start.

As with all oncoplastic surgery, mastectomy should aim to perform as complete an oncological operation as possible. However, maintaining the footprint of the breast (in terms of medial, lateral, superior and inferior levels of dissection) along with healthy mastectomy flaps is essential. When applicable, the use of inframammary mastectomy with implant reconstruction has major aesthetic and safety advantages [10]. Similarly, the vertical mastectomy techniques, with or without nipple preservation can be ideal for autologous free flap reconstruction allowing adequate access for microsurgery whilst maintaining an optimum skin envelope. Limiting dissection to the breast footprint maximises vascular and some nerve supply to the breast skin and minimises the required shaping of a flap reconstruction. Nipple preservation is now considered for all mastectomy and immediate reconstruction cases where oncologically appropriate and confers significant psychosexual benefits [11, 12].

### Oncoplastic Breast Conservation Surgery

Oncoplastic breast surgery aims to achieve good aesthetic outcomes for women with breast cancers who would have unacceptable outcomes with other BCS techniques, and in addition, enable breast-conserving surgery for larger breast cancers. Thus, many women who are treated by oncoplastic breast surgery would otherwise have had a poor aesthetic outcome from standard techniques of BCS or have been recommended mastectomy. For many women oncoplastic breast conserving surgery offers the best, simplest, lowest risk, and sometimes only option for a good aesthetic and practical outcome of breast cancer surgery.

An oncoplastic procedure aims to minimise cosmetic detriment to the breast by eliminating surgical cavities that will then create distortion, hence, the terms parenchymal redistribution or parenchymal replacement have been used. Our own term, therapeutic mammaplasty, covers all forms of reduction and mastopexy techniques, but in practice it can be easier to consider these separately. Therefore, broadly speaking, breast-conserving surgical techniques fall into four main categories:

- ○ Simple wide local excision
- ○ Therapeutic breast reduction
- ○ Therapeutic mastopexy
- ○ Volume replacement.

**Fig. 1:**
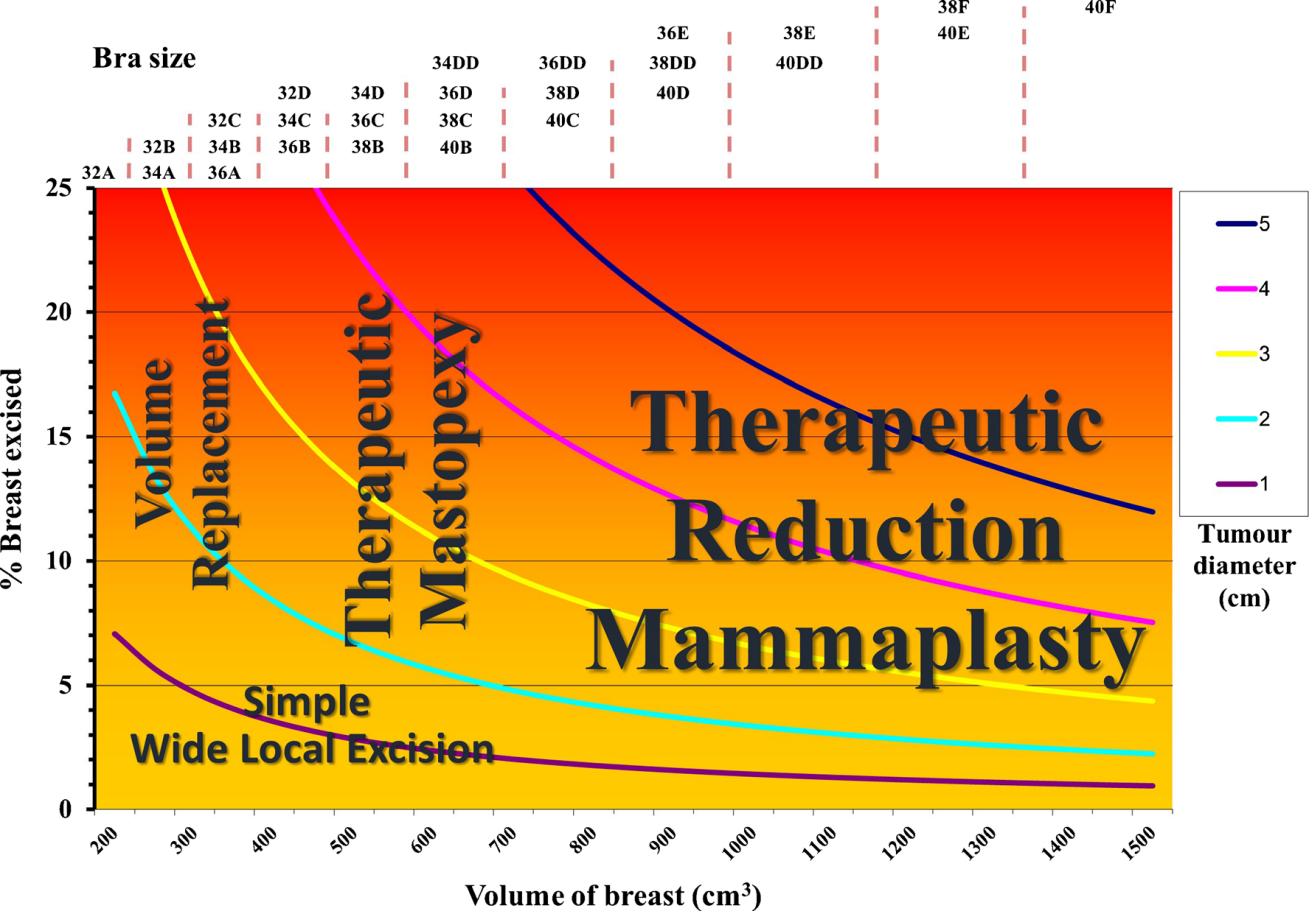
The graph illustrates the role of the four main techniques in oncoplastic breast-conserving surgery according to breast size (volume and bra size), tumour size and estimated percentage of breast volume that would be removed as a wide local excision

The role of these individual techniques in breasts of different sizes is illustrated in Fig. 1.

Poor cosmetic outcome after simple wide local excision is perhaps best predicted by the percentage of breast tissue being removed and the location of the breast cancer although many factors contribute. We have previously correlated the percentage of breast tissue removed with patient satisfaction in a study performed on women who did not have oncoplastic breast-conserving surgery and have shown that depending on tumour location, percentage excisions over 5–15 % are generally associated with an unsatisfactory outcome if oncoplastic surgery is not used [13]. Nowadays, although we would consider that all breast surgery should be oncoplastic, there are simple and complex forms of oncoplastic surgery.

For those whose practice includes screen-detected disease, there may be many indications to keep the surgery very simple, with minimum undermining of surrounding tissue and closure of defect. The main oncoplastic requirement here would be thoughtful incision planning, an understanding of how breast deformity occurs in order to avoid it and careful tissue handling. Any oncological cavity within the breast will collapse and pull both parenchyma and skin towards the cavity. Any skin excision or even incision will contract and create some distortion. Central, medial cavities have relatively less laxity and volume so they create more deformity. For example, a transverse skin incision, or even worse excision, in the inferior pole of the breast will pull down the nipple, create breast ‘beaking’ and poor cosmetic outcomes. By orienting the incision vertically, the natural lateral laxity of the breast is used to fill the defect and the nipple position is maintained. Scars around the areola and at the lateral or inferior breast crease can be employed in suitable cases to avoid scar visibility. The use of skin undermining with simple wide local excision can allow greater parenchymal mobility when the tumour defect does not easily collapse. However, extensive skin or parenchymal undermining are often more disruptive and at times unpredictable compared to a formal oncoplastic procedure as described below. Skin undermining by itself is more acceptable but if combined with parenchymal undermining can create significant disruption, fat necrosis and deformity.

For women with large breasts, particularly those with morbidity associated with breast size, bilateral breast reduction should, in our opinion, always be offered when breast reduction skills are available and of a high standard. This is a good option for any such woman wishing or accepting of a reduction, with any cancer size deemed suitable for breast conservation including those with very small cancers. Breast reduction can reduce the additional morbidity from radiotherapy, as well as achieve a quality of life benefit [14–19]. Breast reduction also reduces risk of subsequent breast cancer [20]. For those with larger cancers, a significant reduction can enable breast conservation and be a particularly attractive option when compared to mastectomy and reconstruction in such cases. In this category of procedure therefore, women are undergoing a significant reduction in overall breast size with a large volume of normal breast tissue being removed in addition to the wide local excision. The breast shape is usually maintained by creating secondary pedicles, in addition to the nipple pedicle [5, 21]. There are, however, many ways of achieving an acceptable breast shape, and not every large-breasted woman with breast cancer is a ideal candidate for a standard technique of breast reduction. In such high risk cases, simplified forms of breast reduction can safely achieve the same aims if a woman is accepting of the associated scarring, and breast reduction in high risk cases becomes particularly simple if a woman is accepting of having her nipples removed as part of the resection [22]. These techniques include standard vertical or Wise pattern reductions but without any nipple or parenchymal pedicles at all. They are suitable for cancers that lie within these excision sites, and as they have no skin or parenchymal undermining or any pedicles are very safe and effective. Even simpler are the transverse ellipse ‘Melon slice’ excisions that may or may not excise the nipple but retain enough breast tissue to achieve an acceptable breast mound and shape.

For women with ptotic breasts who do not necessarily wish a breast reduction, but are accepting of an alteration in breast shape, bilateral therapeutic mastopexy is often the procedure of choice. The principle here is that the only breast volume reduction is the wide local excision itself. Hence, there is usually only a small overall reduction in breast volume but a variable reduction in the skin envelope, with the procedure being more akin to a mastopexy than a reduction [23]. An improved breast aesthetic may be the additional benefit. There are a wide variety of techniques described that would fulfil the objectives of this category of procedure, such as a tennis racket [8], Benelli [24] or batwing mammaplasty [25]. Our preference would be to use a vertical scar mastopexy and whole or hemi-breast rotations [23]. In each of these operative procedures, nipple position and breast shape are altered and the skin envelope is reduced to a varying degree. In some cases, a woman may be accepting of having just the affected breast adjusted and no symmetrising procedure. This is often the best option when the breast shape is only being altered to a small degree, one of the principles of oncoplastic surgery being that preserving breast shape and avoiding deformity trumps overall symmetry as a priority outcome.

The use of this range of therapeutic mammaplasty techniques is dependent on experience in both planning and execution of the techniques, and it is obviously vital that a surgeon gets minimal complications with standard mammoplasty techniques before applying them to a cancer setting. It is very possible to minimise complications with careful planning and patient selection, avoiding tension in the skin closure and careful dissection of the parenchymal pedicles with a good understanding of vascularity to avoid fat necrosis and infections. When the skills are not available, it will be better to use alternative simpler techniques with direct access to the cancer.

For women with small or non-ptotic breasts, breast form is usually best maintained by combining the wide local excision with volume replacement. The LICAP, MICAP and AICAP (lateral, medial and anterior intercostal artery perforators) flaps along with the LTAP (lateral thoracic artery perforator) flap provide versatile local means of volume replacement with acceptable donor site scarring. Although the most commonly used LICAP flap is primarily suitable for lateral-based tumours, in our opinion, there are very few cases with this breast morphology regardless of tumour location that are not suitable for a local perforator flap [7, 26, 27]. The MICAP and AICAP (medial and anterior intercostal artery perforator) flaps are used for more medially based tumours. In those requiring a larger volume flap or needing even greater reach, a TAP (thoracodorsal artery perforator) flap may occasionally be used. Other methods of volume replacement include the latissimus dorsi miniflap, the omental flap, upper abdominal advancement flaps, immediate fat grafting and free flap techniques such as the TUG (transverse upper gracillis) flap [28–32].

In summary therefore, a sensible approach is to keep it simple where possible, particularly in those women with very small cancers. Breast reduction can and should be offered to all women with breast cancer and very large breasts. Therapeutic breast reduction and therapeutic mastopexy (collectively termed therapeutic mammaplasty) both give the option of maintaining a good breast shape and symmetry in the range of breast size that we most commonly encounter. Volume replacement with a local perforator flap is usually the best option for small, non-ptotic breasts. With appropriate minor modification, the four categories of procedure described are each capable of managing a wide local excision defect in any part of the breast. Importantly, oncoplastic surgery caters for the full range of breast size and shape, not just the larger breast. It allows for generous margins of excision, which translate into low rates of margin involvement and second therapeutic procedures [23]. Oncoplastic surgery would almost always be performed at one operation with simultaneous symmetrising reduction/mastopexy or volume replacement as appropriate to the category of technique being used. There is rarely any benefit in delaying a symmetrising procedure if one is desired. Delayed symmetrisation is not easier and not more predictable, and it obviously has the potential to leave women with significant asymmetry for a variable period of time. In addition, delaying volume replacement makes the procedure more difficult and more likely to require skin-bearing flaps.

In general, when different options are available, the simplest is preferred. Many women are accepting of small indentations and asymmetries that surgeons would not be ‘proud of’, and the primary focus and aim should always be to achieve a successful oncological outcome and not necessarily a ‘perfect’ breast. However, oncoplastic surgery allows appropriate cancer surgery to be combined with an acceptable aesthetic outcome, in some cases even an enhanced aesthetic outcome.

In some situations, oncoplastic breast-conserving surgery allows women the choice of avoiding mastectomy. This may not only be particularly relevant to those who would require post-mastectomy radiotherapy but also to those who require axillary node clearance and those with morbidities and risk factors for bigger surgery; all of which would be potential exclusion criteria or high risk factors for immediate breast reconstruction [23, 33, 34].

### Oncocosmetic Surgery

In all published series of breast-conserving surgery, aesthetic outcome is reported in terms of the degree to which the outcome is worse than the starting point. The perceived pinnacle of aesthetic outcome is therefore the maintenance of existing breast form or it being slightly worse, and these two categories are usually combined in reports and achieved in approximately 60–85 % of BCS cases for small cancers and approximately 55–70 % of mastectomy with reconstruction cases. A question that is not often asked before breast cancer surgery is what a woman thinks of her breast size and shape before surgery. If the answer is negative, then maintaining breast form may not be as ideal a result as the surgeon imagines. Such cases require additional consideration, and the surgery that should be considered may fall into the controversial category of oncocosmetic.

Although still debatable for many, the use of breast reduction for those who desire smaller breasts when being treated for cancer is in our opinion easy to support. In the case of large breast size and therapeutic reduction mammaplasty, it is easy to imagine how a woman may desire a reduced breast size, how this may lend itself to a wide local excision and how she may positively perceive the outcome of her surgery. In many cases, the reduction has enabled breast conservation as an option. However, in other cases, for example a 10-mm cancer, the reduction is performed for quality of life and cosmetic benefit, as well as perhaps to avoid the potential morbidity of radiotherapy.

For smaller breasts, breast conservation can maintain aesthetic appearance but rarely improves it. For those with ptosis, a therapeutic mastopexy as described above may achieve an optimised aesthetic for some. In reality, for most cases, it will be used to minimise cosmetic deformity, which smaller breasts are more susceptible to.

For others, a larger breast size may be the desire, and for such women a mastectomy and augmented reconstruction with contralateral augmentation, or bilateral mastectomy and augmented reconstruction may be an option to consider. Adjustment in overall breast size is a relatively frequent outcome in bilateral mastectomy and breast reconstruction, and achieving positive aesthetic outcomes rather than just maintaining breast form or avoiding deformity should be within the skill set of an oncoplastic surgeon. This discussion may raise eyebrows for some, and it would be important to appreciate that oncocosmetic surgery would only be appropriate for highly selected cases and, in addition, experience in pure cosmetic breast surgery would be essential for those who would practice it. However, as standards of aesthetic outcome of breast cancer surgery have been raised, there is a greater realisation of the importance of aesthetic outcome for some women and a greater understanding of the effect of breast cancer surgery and adjuvant treatments on body image and psychosocial outcomes [35, 36]. Importantly, women with breast cancer never wanted the surgery required to remove their disease. The oncoplastic surgery that they may choose to correct their appearance as a result of this may have the option of not just restoring original form to a varying degree but may, with little or no extra cost, be able to offer a more desirable outcome. Both patient and surgeon should feel this is a worthwhile endeavour.

### Conclusions

The principles of oncoplastic surgery are applicable to all breast cancer surgery, although the options it can offer will have much more relevance to some women than others. The primary aim is always disease eradication, but the physical effects of this can and should be minimised. A range of techniques is possible spanning from very simple and functional, to complex and cosmetic, but women who may benefit can only do so if it is offered. Much of what is discussed in this article reflects an individualised care to breast cancer surgery, far removed from the one of two options approach of old.

## References

[1] Audretsch W, Rezai M, Kolotas C (1998). Tumour-specific immediate reconstruction in breast cancer patients. Semin Plastic Surg.

[2] Petit JY, Rigaut L, Zekri A, Le M (1989). Poor esthetic results after conservative treatment of breast cancer. Technics of partial breast reconstruction. [French]. Ann Chir Plast Esthet.

[3] Clough KB, Kroll SS, Audretsch W (1999). An approach to the repair of partial mastectomy defects. Plastic & Reconstructive Surgery.

[4] Clough KB, Lewis JS, Couturaud B (2003). Oncoplastic techniques allow extensive resections for breast-conserving therapy of breast carcinomas. Ann Surg.

[5] McCulley SJ, Macmillan RD (2005). Planning and use of therapeutic mammoplasty—Nottingham approach. Br J Plast Surg.

[6] Rainsbury RM (2007). Oncoplastic breast-conserving reconstruction: indications, benefits, choices and outcomes. Nat Clin Pract Oncol.

[7] Hamdi M, Van Landuyt K, de Frene B, Roche N, Blondeel P, Monstrey S (2006). The versatility of the inter-costal artery perforator (ICAP) flaps. J Plast Reconstr Aesthet Surg.

[8] Clough KB, Kaufman GJ, Nos C, Buccimazza I, Sarfati IM (2010). Improving breast cancer surgery: a classification and quadrant per quadrant atlas for oncoplastic surgery. Ann Surg Oncol.

[9] Macmillan RD. Mastectomy for Breast Cancer: Tips and Pitfalls. In Companion to Specialist Surgical Practices. Ed Dixon M. 2014.

[10] Endara M, Chen D, Verma K, Nahabedian MY, Spear SL (2013). Breast reconstruction following nipple-sparing mastectomy: a systematic review of the literature with pooled analysis. Plast and Reconstr Surg.

[11] Wei CH, Scott AM, Price AN, Miller HC, Klassen AF, Jhanwar SM (2016). Psychosocial and sexual well-being following nipple-sparing mastectomy and reconstruction. Breast J.

[12] Didier F, Radice D, Gandini S, Bedolis R, Rotmensz N, Maldifassi A (2009). Does nipple preservation in mastectomy improve satisfaction with cosmetic results, psychological adjustment, body image and sexuality?. Breast Cancer Res Treat.

[13] Cochrane RA, Valassiadou P, Wilson ARM, Al-Ghazal SK, Macmillan RD (2003). Cosmesis and satisfaction after breast-conserving surgery correlates with the percentage of breast volume excised. Br J Surg.

[14] Lyngholm CD, Christiansen PM, Damsgaard TE, Overgaard J (2013). Long-term follow-up of late morbidity, cosmetic outcome and body image after breast conserving therapy. A study from the Danish Breast Cancer Cooperative Group (DBCG). Acta Oncol.

[15] Waljee JF, Hu ES, Ubel PA, Smith DM, Newman LA, Alderman AK (2008). Effect of esthetic outcome after breast-conserving surgery on psychosocial functioning and quality of life. J Clin Oncol.

[16] Al-Ghazal SK, Blamey RW, Stewart J, Morgan AAL (1999). The cosmetic outcome in early breast cancer treated with breast conservation. Eur J Surg Oncol.

[17] Moody AM, Mayles WPM, Bliss JM (1994). The influence of breast size on late radiation effects and association with radiotherapy dose inhomogeneity. Radiother Oncol.

[18] Neal AJ, Torr M, Helyer S, Yarnold JR (1995). Correlation of breast dose heterogeneity with breast size using 3D CT planning and dose volume histograms. Radiother Oncol.

[19] Goldsmith C, Haviland J, Tsang Y (2011). Large breast size as a risk factor for late adverse effects of breast radiotherapy: is residual dose inhomogeneity, despite 3D treatment planning and delivery, the main explanation?. Radiother Oncol.

[20] Palmieri B, Benuzzi G, Costa A, Grappolini S (2006). Breast reduction and subsequent cancer: a prophylactic perspective. Breast.

[21] Macmillan RD, Chan CW, McCulley SJ. Volume displacement techniques

[22] James R, McCulley SJ, Macmillan RD (2015). Oncoplastic and Reconstructive Surgery in the Elderly. Br J Surg.

[23] Macmillan RD, James R, Gale KL, McCulley SJ (2014). Therapeutic mammaplasty. J Surg Oncol.

[24] Benelli L (1990). A new periareolar mammaplasty: the “round block” technique. Aesthetic Plast Surg.

[25] Silverstein MJ, Mai T, Savalia N, Vaince F, Guerra L (2014). Oncoplastic breast conservation surgery: the new paradigm. J Surg Oncol.

[26] Hamdi M, Spano A, Van Landuyt K, D’Herde K, Blondeel P, Monstrey S (2008). The lateral intercostal artery perforators: anatomical study and clinical application in breast surgery. Plast Reconstr Surg.

[27] McCulley SJ, Schaverien MV, Tan VK, Macmillan RD (2015). Lateral thoracic artery perforator (LTAP) flap in partial breast reconstruction. J Plast Reconstr Aesthet Surg.

[28] Rusby JE, Paramanathan N, Laws SA, Rainsbury RM (2008). Immediate latissimus dorsi miniflap volume replacement for partial mastectomy: use of intra-operative frozen sections to confirm negative margins. Am J Surg.

[29] Zaha H, Onomura M, Nomura H, Umekawa K, Oki M, Asato H (2012). Free omental flap for partial breast reconstruction after breast-conserving surgery. Plast Reconstr Surg.

[30] Ogaw T, Hanamura N, Yamashita M, Ito M, Kimura H, Nakamura T (2013). Abdominal advancement flap as oncoplastic breast conservation: report of seven cases and their cosmetic results. J Breast Cancer.

[31] Biazus JV, Falcão CC, Parizotto AC, Stumpf CC, Cavalheiro JA, Schuh F (2015). Immediate Reconstruction with Autologous fat Transfer Following Breast-Conserving Surgery. Breast J.

[32] McCulley SJ, Macmillan RD, Rasheed T (2011). Transverse Upper Gracilis (TUG) flap for volume replacement in breast conserving surgery for medial breast tumours in small to medium sized breasts. J Plast Reconstr Aesthet Surg.

[33] Peled AW, Sbitany H, Foster RD, Esserman LJ (2014). Oncoplastic mammoplasty as a strategy for reducing reconstructive complications associated with postmastectomy radiation therapy. Breast J.

[34] Wang F, Peled AW, Chin R, Fowble B, Alvarado M, Ewing C (2016). The impact of radiation therapy, lymph node dissection, and hormonal therapy on outcomes of tissue expander—implant exchange in prosthetic breast reconstruction. Plast Reconstr Surg.

[35] Al-Ghazal SK, Fallowfield L, Blamey RW (1999). Does cosmetic outcome from treatment of primary breast cancer influence psychosocial morbidity?. Eur J Surg Oncol.

[36] Deshields TL, Reschke A, Walker MS, Brewer A, Taylor M (2007). Psychological status at time of diagnosis and patients’ ratings of cosmesis following radiation therapy for breast cancer. J Psychosoc Oncol.

